# Genome-Wide Association Studies to Identify Loci and Candidate Genes Controlling Kernel Weight and Length in a Historical United States Wheat Population

**DOI:** 10.3389/fpls.2018.01045

**Published:** 2018-08-03

**Authors:** Sintayehu D. Daba, Priyanka Tyagi, Gina Brown-Guedira, Mohsen Mohammadi

**Affiliations:** ^1^Department of Agronomy, Purdue University, West Lafayette, IN, United States; ^2^Department of Crop and Soil Sciences, North Carolina State University, Raleigh, NC, United States; ^3^Small Grains Genotyping Laboratory, United States Department of Agriculture, Agricultural Research Services, Raleigh, NC, United States

**Keywords:** kernel weight, kernel length, QTL, GWAS, candidate gene, historical germplasm

## Abstract

Although kernel weight (KW) is a major component of grain yield, its contribution to yield genetic gain during breeding history has been minimal. This highlights an untapped potential for further increases in yield via improving KW. We investigated variation and genetics of KW and kernel length (KL) via genome-wide association studies (GWAS) using a historical and contemporary soft red winter wheat population representing 200 years of selection and breeding history in the United States. The observed changes of KW and KL over time did not show any conclusive trend. The population showed a structure, which was mainly explained by the time and location of germplasm development. Cluster sharing by germplasm from more than one breeding population was suggestive of episodes of germplasm exchange. Using 2 years of field-based phenotyping, we detected 26 quantitative trait loci (QTL) for KW and 27 QTL for KL with *–log_10_(p)* > 3.5. The search for candidate genes near the QTL on the wheat genome version IWGSCv1.0 has resulted in over 500 genes. The predicted functions of several of these genes are related to kernel development, photosynthesis, sucrose and starch synthesis, and assimilate remobilization and transport. We also evaluated the effect of allelic polymorphism of genes previously reported for KW and KL by using Kompetitive Allele Specific PCR (KASP) markers. Only *TaGW2* showed significant association with KW. Two genes, i.e., *TaSus2-2B* and *TaGS-D1* showed significant association with KL. Further physiological studies are needed to decipher the involvement of these genes in KW and KL development.

## Introduction

Yield genetic gains in wheat slowed down over the last two decades (Brisson et al., 2010; Lin and Huybers, 2012; Ray et al., 2012), threatening world food security. Simmonds et al. (2014) highlighted that grain number (GN) per unit area and kernel weight (KW) are main determinants of grain yield (GY). These two traits, i.e., GN and KW together represent total sink-strength in wheat. Over the course of the breeding history of cereals, the per unit area GN has considerably increased, while KW showed no significant increase or even decreased slightly (Brancourt-Hulmel et al., 2003; Carver, 2009). KW is determined by kernel size, which is a function of kernel width, length, and thickness, and degree of grain filling (Simmonds et al., 2014; Su et al., 2016). Though complex, KW is the most heritable trait among yield components (Su et al., 2016), with heritability reaching as high as 0.87 (Bergman et al., 2000; Wiersma et al., 2001). Kernel development in wheat involves cell division, water uptake, accumulation of starch and protein, maturation, and desiccation (Altenbach and Kothari, 2004). While grain expansion enforced by endosperm cell division and water uptake are components of sink-strength, assimilate (e.g., starch) supply (Emes et al., 2003) through current photosynthesis or remobilization of reserves from vegetative tissues (Bidinger et al., 1977; Schnyder, 1993; Gebbing and Schnyder, 1999) are components of source-strength.

Several QTL for KW and kernel dimension traits have been localized across the 21 wheat chromosomes (Zhang et al., 2012; Jaiswal et al., 2015; Chen et al., 2016). Only a few loci were functionally validated in wheat, compared to other cereals such as rice for KW and dimension traits, due to the lack of reference genome sequence and ploidy complexity (allohexaploid, 2*n* = 6X = 42) of the wheat (Simmonds et al., 2014; Su et al., 2016). To this end, several genes identified in other cereals were postulated to be involved in kernel trait determination in wheat. *Sucrose transporter TaSUT* was shown to regulate the translocation of assimilates from source to sink tissues (Aoki et al., 2004; Deol et al., 2013). *Sucrose synthase TaSus* catalyzes the first step in the conversion of sucrose to starch, particularly the conversion of sucrose to fructose and *UDP-glucose* (Jiang et al., 2011; Hou et al., 2014). Cytokinin oxidase *TaCKX* which inactivates *cytokinin* reversibly was shown to have an effect on KW (Zhang et al., 2010; Lu et al., 2015; Chang et al., 2016). *Cytokinin* oxidase such as TaCKX1 highly expressed during early seed development (Song et al., 2012). *Cell wall invertase TaCWI* exerts a role in kernel size control by sink tissue development and carbon partitioning (Ma et al., 2012). Several other grain size related genes include *TaGS-D1* which codes for *Glutamine synthase* with effect on KW and kernel length (KL) (Zhang et al., 2014); *TaGW6*, which encodes for *indole-3-acetic acid (IAA)-glucose hydrolase* (Hu et al., 2016); and *TaGW2* (Pflieger et al., 2001; Su et al., 2011; Bednarek et al., 2012) encodes for a *RING-type protein with E3 ubiquitin ligase* activity that controls KW and interestingly, positively regulates grain size as opposed to the rice *GW2* gene which has negative effect on grain size (Bednarek et al., 2012). Deployment and transferability of these genes in populations and environments beyond the discovery populations and environments is a valuable applied research question.

Genome-wide association studies (GWAS) that dissect the genetic basis of traits and propose candidate genes (Pflieger et al., 2001), could be an important step for trait improvement. The scope of genes and alleles that are identified in GWAS pipelines depends, to a large extent, on the variation that is in the germplasm. In most cases, discovery panels consist of elite lines from multiple breeding programs (Mohammadi et al., 2015), which usually demonstrate high familial relatedness; or GenBank accessions (Zhao et al., 2011), which are often genetically structured by the geography of origin. The third type of diversity panel could be accessions sampled from adapted breeding materials in a spectrum of time, i.e., from the past to present time, which can identify alleles that became extinct or are recently introduced. Analyses of genetic gain in wheat have not postulated significant improvement in KW parallel to what observed in GN. The quest for increases in KW parallel to GN will depend on the genetic nature of KW that may be gained from a past-to-present perspective of an allelic composition of wheat accessions. Crossing schemes and selections from among segregating progeny, which is a landmark of the breeding process, can be thought as accelerated evolutionary forces that either rapidly fix or purge alleles. Therefore, current elite germplasm is likely unable to depict alleles that contributed in the past or are now fixed. Mapping using in-time diversity panels allows understanding of the realized trends and gain or loss of beneficial alleles, both very important factors for strategizing breeding programs.

Development of molecular markers for KW will greatly facilitate the selection process. In this study, we utilize a unique wheat population composed of historical and contemporary germplasm, representing breeding history and selection of over 200 years in the United States wheat industry. The panel has a considerable variation for several traits including KW, allowing a high power of QTL detection. The objectives of this study include, (1) to identify quantitative trait loci (QTL) for KW and KL in a historical and contemporary set of soft red winter wheat (SRWW) in the United States, (2) to search the recently published wheat reference genome IWGSC RefSeq v1.0 annotation v1.0 to mine candidate genes that are putatively responsible for determination of KW and KL in wheat.

## Materials and Methods

### Plant Materials and Field Trials

Historical and contemporary SRWW cultivars and breeding lines, representing 200 years (1814–2015) of selection and breeding history in diverse geographical regions in the United States were phenotyped. The seed for most of the entries was provided by the National Small Grains Collection (NSGC), United States Department of Agriculture (USDA) in Aberdeen, Idaho. Accessions were field grown to maturity at the Agronomy Center for Research and Education (ACRE), Purdue University, West Lafayette in the cropping seasons of 2015–2016 and 2016–2017. We grow each entry in a 1-m long single row plot with 25 cm row spacing. The crop received 106 kg N ha^-1^ in both years just after the winter dormancy break. As old accessions with no height reducing (*Rht*) genes were at the risk of lodging and disruption of grain filling process, we assembled guards and ropes around row plots to prevent lodging.

### Phenotyping

Each single-row was hand harvested and processed at ACRE. Two kernel characteristics were measured, i.e.; KW and KL. We hand-harvested multiple heads from each entry, oven-dried, and measured the weight of two replicates of 100 kernels. The average KW was then expressed in milligram (mg). The experiments in 2015–2016 and 2016–2017 seasons did not include the same number of entries. In the 2015–2016 season, 265 entries were phenotyped. In the 2016–2017 season, 214 entries were phenotyped. Only 160 entries were in common between 2015–2016 and 2016–2017. Altogether, in both years KW from 325 entries were measured. The KW data of 2015–2016 and 2016–2017 are referred to as KW16 and KW17. The Best Unbiased Linear Predictor (BLUP) of KW across both years with 325 entries is referred to as KW1617. For KL, 265 entries were measured in 2015–2016 and 217 entries were measured in 2016–2017. The common entries between both years were 160. Altogether, in both years KL from 323 entries were measured. For measuring KL, we aligned 10 kernels to the side of a ruler. The resulting measurements were divided by 10 and expressed in millimeter (mm) for a single kernel. Similar to KW, KL data are referred to as KL16, KL17, and KL1617 for 2016, 2017, and combined BLUP estimates, respectively.

### Analysis of Traits and Trends

The relationship between the datasets generated in different environments was used as a measure of repeatability of the phenotypic measurements. Correlations among the different datasets can be indicative of technical heritability and repeatability of KW and KL in diverse environments. We also estimated the broad-sense heritability values for both traits using the variance components. The trend of traits over time was visualized by using boxplots of KW1617 and KL1617 datasets of the four year-groups (YG ≤ 1920, 1920 < YG ≤ 1960, 1960 < YG < 2000, and YG ≥ 2000). The total number of entries in each YG and the number of entries phenotyped for KW and KL in the 2 years are shown in **Table 1**.

**Table 1:** Distribution of the lines in each of the four year-groups and years.

Year-group	Total over 2016 and 2017	Phenotyped	
		2016	2017
Before 1920	35	33	15
1920–1960	64	57	28
1960–2000	168	152	121
After 2000	57	23	50

### Genotyping

For genotyping, we extracted DNA from 15-day-old leaf samples and followed a sequencing-based genotyping procedure explained by Poland et al. (2012). The genomic libraries were created using *Pst1-Msp1* restriction enzyme combinations. The samples were pooled together in 96-plex to create libraries and each library was sequenced on a single lane of Illumina Hi-Seq 2500. SNP calling was performed using the TASSEL5 GBSv2 pipeline^1^ using 64 base kmer length and minimum kmer count of 5. Reads were aligned to the wheat reference “IWGSC_WGAv1.0”^2^ using aln method of Burrows–Wheeler Aligner (BWA) version 0.7.10 (Li and Durbin, 2009). We used the default parameters of BWA. This resulted in 309,711 unfiltered SNP loci. The SNPs not assigned to any chromosome were removed. The remaining markers were filtered for minor allele frequency (MAF) ≥5% and missing values ≤30%, which resulted in 60,132 SNP. Missing data were imputed using the Linkage Disequilibrium K-number neighbor imputation (LDKNNi) (Money et al., 2015) algorithm implemented in Tassel 5.0 (Bradbury et al., 2007). We also estimated the error rates of LDKNNi imputation for the different level of masking and the results are given in **Supplementary Table S1**.

### Population Structure

Population structure was evaluated using principal component analysis (PCA) of 60,132 SNP markers, implemented in TASSEL5.0 (Bradbury et al., 2007). Population structure was then visualized using a three-dimensional plot of the first three principal components (PCs) using the R package “Scatterplot3d” (Ligges and Maechler, 2003). We also conducted model-based Bayesian clustering analysis using Structure 2.3.4 (Pritchard et al., 2000). Total of 16,313 tag SNPs were used for this analysis, which were selected using tagger function in Haploview (Barrett et al., 2005). The parameters in the tagger function set to “pairwise tagging only” with *R*^2^ = 0.8. To infer population structure for 325 wheat genotypes, we ran structure analysis for *K*-values from 2 to 10. Both the length of burn-in period and the number of iterations were set at 10,000. The *K*-value reached a plateau when the minimal number of groups that best described the population sub-structure has been attained (Pritchard et al., 2000). The average *K*-values were plotted against their respective logarithm of the probability of likelihood, i.e., LnP(D). The rate of change in the log probability of data between successive *K*-values (Evanno et al., 2005) was used to predict the most appropriate number of subpopulations. We described the differentiation among the four clusters using fixation index (*F_ST_*) method (Wright, 1951, 1978).

### Genome-Wide Association Studies

Association mapping was performed for the two kernel traits using the 60,132 SNP markers in GAPIT package (Lipka et al., 2012). We used mixed linear model (MLM), applying P3D (Population Parameters Previously Determined) described as Mixed-Model Association on eXpedited (EMMAX) algorithm (Kang et al., 2010). Our model included markers and the first three PCs of the population structure as fixed effects. Kinship as familial relatedness matrix and residual terms were considered as random effects. Manhattan plots were produced using the negative logarithm at base 10 of the *p*-values, shortened as *-log10(p)* using “qqman” package of R (Turner, 2014) across the physical map. The markers with -*log10(p)* > 3.5 were identified for further characterization. We constructed LD block for significant SNP markers within a chromosome using HAPLOVIEW v4.2 (Barrett et al., 2005) to assign markers to short blocks. Changes in favorable alleles over time was evaluated using the same four year-groups that were used for trend analysis. The cumulative effect of identified favorable alleles on the kernel traits was also evaluated.

### Effect of Known Loci/Genes on Kernel Traits

Allelic composition of previously reported loci/genes implicated in kernel traits, i.e., *TaSus2-2B, TaCWI-4A, TaCWI-5D, TGW6, TaTGW6-A1, TaGS-D1, TaGW2, Rht-1B*, and *Rht-1D* were evaluated using KASP markers described in Rasheed et al. (2016). These polymorphisms were used in a Student’s *t*-test to statistically assess the effect of each known locus/gene on the variation of kernel traits.

### Candidate Gene Identification

We retrieved high confidence wheat genes surrounding (within ±250 kb) representative SNPs for the genomic regions identified both for KW and KL. For gene search purpose, we used IWGSC RefSeq v1.0 annotation v1.0, iwgsc_refseqv1.0_HighConf_2017Mar13.gff3.zip ^3^.

## Results

### Phenotypic Variation

**FIGURE 1:**
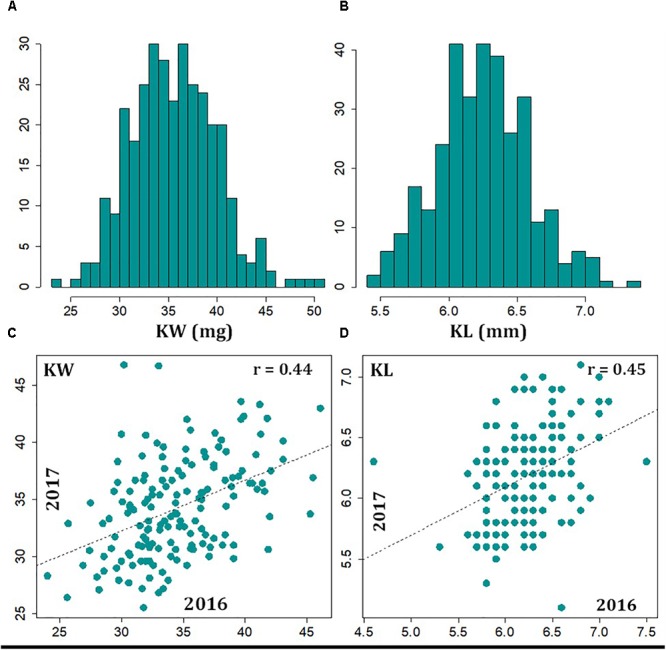
Distribution of 2-year BLUP values kernel weight **(A)** and kernel length **(B)**, and correlations, as evidence of technical repeatability, observed between 2016 and 2017 data for kernel weight **(C)** and kernel length **(D)**.

We evaluated the variation of KW and KL in a historical and contemporary collection of cultivars and experimental breeding lines, representing 200 years of breeding and selection history. Across the 2 years of study, the Best Linear Unbiased Estimate (BLUE) values (i.e., KW1617) showed a mean of 35.6 mg with a range from 23.5 to 50.6 mg (**Figure 1A**). The 20 greatest KW entries showed an average of 44.8 ± 2.5 mg and the 20 smallest KW entries showed an average of 27.7 ± 1.4 mg. The mean phenotype value for KW16 and KW17 were 35.2 mg (with a range of 23.3–50.7 mg) and 35.2 mg (with a range of 25.5 –49.8 mg), respectively (Supplementary Figure S1). The mean of KL1617 BLUE values was 6.3 mm, with a range of 5.3–7.4 mm (**Figure 1B**). The 20 longest kernel entries showed an average of 7.0 ± 0.15 mm and the 20 shortest kernel entries showed an average of 5.6 ± 0.07 mm. The mean phenotype value for KL16 and KL17 were 6.3 mm (with a range of 4.6–7.5 mm) and 6.2 mm (with a range of 5.1–7.1 mm), respectively (Supplementary Figure S1).

The correlation of traits among the different environments can be used as a measure of repeatability. Using the common entries between the 2 years, a moderate correlation (*r* = 0.44, *p*-value < 0.01) was observed for KW between the 2 years (**Figure 1C**). Similarly, we observed moderate correlation (*r* = 0.45, *p*-value < 0.01) between KL measurements from the 2 years (**Figure 1D**). The broad-sense heritability for both KW and KL, based on measurements in the 2 years, turned out to be 0.61 and 0.55, respectively. The correlation of data between the 2 years and measures of heritability suggests that both KW and KL are reasonably stable traits across years. The correlation of KW and KL BLUP values across 323 lines over 2 years was *r* = 0.20 (Supplementary Figure S2).

One of the claims about GY and KW in wheat breeding and selection history is that KW showed no significant increase or even decreased slightly while GY increased (Brancourt-Hulmel et al., 2003; Carver, 2009). Thus, one of our objectives was to investigate whether selection and breeding have increased or decreased kernel traits over the course of breeding history. Overall, the trend for KW was not consistent for the years across the four year-groups (Supplementary Figure S3). Though non-significant, for example, KW16 showed a slightly decreasing trend, with a mean of 36.1 mg across the entries developed before 1920 while 34.6 mg for entries developed after 2000. On the contrary, KW17 showed an increasing trend, with a mean of 33.4 mg before 1920 and a mean of 38.0 mg after 2000. The discrepancy of the trend between 2016 and 2017 could be due to an overrepresentation of Purdue-bred lines in the 2017 trial. The added Purdue lines (*N* = 35) exhibited greater KW (mean of 40.5 g), causing an increasing trend. KL16 remained unchanged over time while KL17 increased until 1960 then dropped afterward (Supplementary Figure S3).

### Population Structure

**FIGURE 2:**
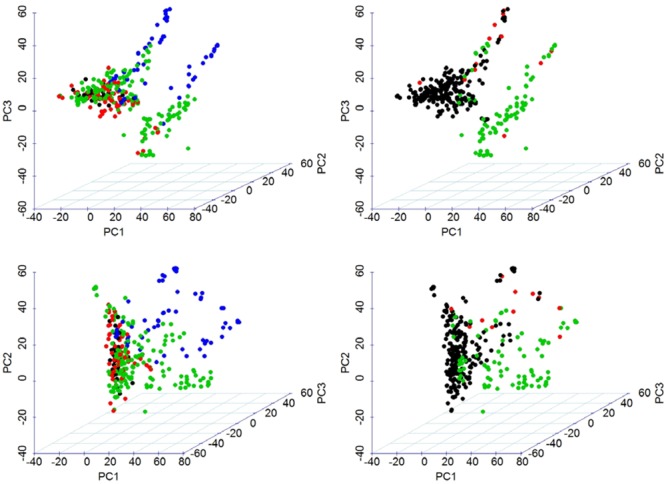
Visualization of population stratification using three-dimensional scatter plot of the first three PCs estimated from 60,134 SNP markers. Top panel shows PC1, PC2, and PC3 in *x*-, *y*-, and *z*-axis configuration. Bottom panel shows PC1, PC3, and PC2 in *x*-, *y*-, and *z*-axis configuration. This allows a more-informed visualization. The left panel shows grouping by year-group and the right panel shows grouping by TaSus2-2B representing 2B.2G translocation from *T. timopheevii*.

We used all the 60,132 SNP markers in the analysis of population structure using PCA. The A, B, and D sub-genomes were represented by 35%, 44%, and 21% of SNPs, respectively. The first three PCs of marker data, altogether, explained 15.0% of the total variation and were used to draw a 3D-plot of the population structure. PC1 clearly grouped the germplasm based on the era of development, i.e., after or before 2000 (**Figure 2**). We also make the grouping for 3D-plot based on 2B.2G translocation form *T. timopheevii* represented by *TaSus2-2B* (**Figure 2**). The result revealed that the panel of 324 genotypes was clustered clearly into two groups, i.e., possessing or not possessing the 2B.2G translocation. The variation in this translocation is also reflected in the values of the PC1.

**FIGURE 3:**
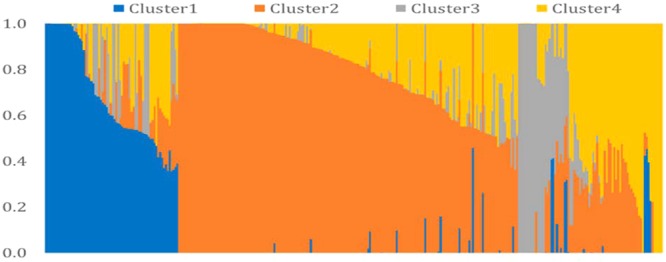
Model-based cluster analysis using 16,313 tag SNPs selected by Haploview software.

We performed model-based clustering using 16,313 tag SNPs, selected using the tagger function of Haploview (Barrett et al., 2005) with the parameters of “pairwise tagging only” and *R*^2^= 0.8. The result from this analysis revealed four sub-populations (**Figure 3**). The number of the entries assigned to each cluster ranged from 28 in Cluster3 to 177 in Cluster2. The detail descriptions of cluster membership is given in **Supplementary Table S2**. In total, 42.9% of the entries were developed by the Purdue Small Grains Breeding Program and therefore, membership of Purdue lines in all clusters is expected. Year of release and geographical region explained group membership partially. For example, Cluster1 was predominantly represented by germplasm developed before 1960 (91.4%) and Cluster2 was predominantly represented by germplasm developed before 2000 (93.8%). A majority (82.1%) of the entries in Cluster3 were developed after 2000. Cluster4 was mainly comprised of genotypes developed between 1920 and 2000. Cluster-sharing among entries originated in the different breeding programs could be an evidence of historical and recent germplasm exchange among breeding programs.

The differentiation among the four clusters and the four year-groups was assessed using the *F_ST_*. The *F_ST_* estimates for pairwise clusters revealed varied levels of allelic differentiation among the four clusters (Supplementary Figure S4). The Cluster3 was differentiated more from the other three clusters, with several of the SNP loci showing *F_ST_* > 0.15 (Wright, 1978). Among the four clusters generated by the model-based analysis, a total of 457 SNP loci out of 60,132 showed significant *F_ST_* (>0.15), indicating allelic differentiation. The majority of significant differentiations were observed between Cluster3 and Cluster4 (224 SNPs), followed by the comparison between Cluster1 and Cluster3, which yielded 215 significant (*F_ST_* > 0.15) SNPs. The comparison between Cluster2 and Cluster3 yielded 102 significant SNPs. The least differentiated clusters were Cluster1 and Cluster2 with all the SNP loci showing a *F_ST_* below 0.15.

### GWAS and Allele Frequency Changes Over Time for KW

**FIGURE 4:**
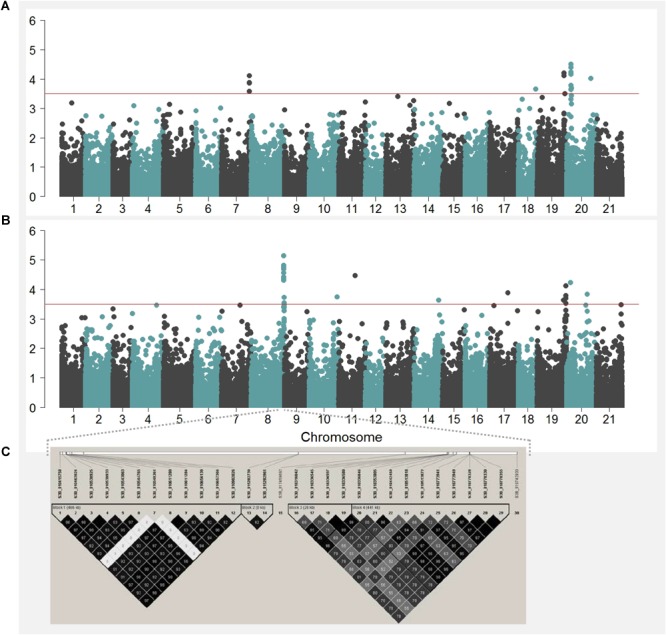
Manhattan plots showing negative log *p*-values of SNPs tested across the 21 chromosomes (i.e., 1 = 1A, 2 = 1B, 3 = 1D, …, 20 = 7B, and 21 = 7D) obtained from GWAS by using 2-year BLUP values for kernel length **(A)** and kernel weight **(B)**. The haplotype blocks estimated for the 30 SNP markers located on the region significantly associated with kernel weight on chromosome 3B is shown in **(C)**.

Any QTL in an individual year or combined 2-year analysis with *–log10(p)* > 3.5 was considered for further discussion. GWAS has resulted in 77 QTL for KW (**Figure 4B**, Supplementary Figure S5, and **Supplementary Table S3**), of which, 30 QTL were stacked in seemingly one genomic location on chromosome 3B. A pair-wise LD criterion of *R*^2^ ≥ 0.75 resolved all 30 QTL on 3B clustered into six LD block regions, with a minimum of one SNP to a maximum of 12 SNP markers per LD block (**Figure 4C**). A similar short-range LD block characterization for all the chromosomes, following *R*^2^ ≥ 0.75, enabled us to assign the 67 QTL to 26 genomic regions (**Supplementary Table S4**) distributed on chromosomes 1B, 2A, 2B, 3B, 4A, 4B, 5A, 6B, 7A, and 7B. Each of these regions was represented with a single SNP with the highest *-log10(p)*.

The highest *–log10(p)* value for KW was for a marker on chromosome 7B, designated as *QKWpur-7B.1* with *–log10(p)* of 5.4 and 4.5 in KW16 and KW1617, respectively. This marker explained 8.3% of phenotypic variation in 2016 with a marker effect of 0.9 mg. Out of 26 QTL identified for KW, 13 represented signals detected in 2016 (four of them also detected in the combined 2-year analysis). These 13 QTL detected for KW16 individually explained a low of 5.1% to a high of 8.3% of the variation in KW16. For KW17, eight genomic regions were identified (one of them also detected in the combined 2-year analysis). Individually, these eight QTL explained from a low of 5.5% to a high of 9.0% of the variation in KW17. Combined 2-year analysis revealed five unique QTL in addition to the four overlapping QTL of KW16 and one overlapping QTL of KW17. These 10 QTL for the combined 2-year data accounted for a low of 3.7% to a high of 5.0% to the phenotypic variation in KW1617.

We were interested in evaluating the frequency of favorable alleles in the identified loci. Out of 26 loci, 13 showed lower than 50% and 13 showed higher than 50% frequency for the favorable alleles. The trend of these allele frequency changes was given only for a subset of loci across year-groups in Supplementary Figure S6. When evaluated over the four year-groups, the frequency of favorable alleles decreased in 18 out of 26 of identified loci. The frequency of favorable alleles increased in four loci. For the remaining loci, it did not show a clear trend.

### GWAS and Allele Frequency Changes Over Time for KL

We considered any QTL in an individual year or combined 2-year analysis with *–log10(p)* > 3.5 as significant and discussed further. GWAS has resulted in 45 QTL for KL (**Figure 4A**, Supplementary Figure S5, and **Supplementary Table S5**). With short-range LD block characterization for all the chromosomes, with criteria of considering SNPs with *R*^2^ ≥ 0.75 in one LD block, we assigned the 45 QTL to 27 genomic regions (**Supplementary Table S6**) distributed on chromosomes 1A, 1B, 2A, 2B, 2D, 3A, 3B, 4D, 6A, 6B, 7A, 7B, and 7D. Each genomic region was represented with a single SNP with the highest *-log10(p)*. The highest *–log10(p)* value for KL was for a marker on chromosome 7B, designated as *QKLpur-7B.3* with *–log10(p)* of 4.5 in KL1617. This genomic region explained 4.8% of phenotypic variation in KL1617 with a marker effect of 0.05 mm. Eleven of the genomic regions were detected in 2016, with three of them also detected in the combined 2-year analysis. These 11 QTL detected for KL16 individually explained from a low of 4.7% to a high of 6.1% of the variation in KL16. For KL17, eight genomic regions were identified, with individual QTL explaining a low of 5.5% to a high of 6.1% of the variation in KW17. The combined analysis revealed eight unique QTL in addition to the three overlapping QTL of KL16. These 11 genomic regions identified for KL1617 accounted from a low of 3.6% to a high of 4.8% to the phenotypic variation in KW1617.

The trend of these allele frequency changes was given only for a subset of loci across the YG in Supplementary Figure S7. Of the 27 loci, seven were higher than 50% in favorable allele frequency while the remaining loci were lower than 50% for favorable allele frequency (data not shown). Fourteen loci showed a decrease in frequency of favorable alleles across the four year-groups. Six loci exhibited an increasing trend of favorable allele across the four year-groups. The remaining seven loci did not show a clear trend across the four year-groups.

### Cumulative Effect of Identified Loci on KW

**FIGURE 5:**
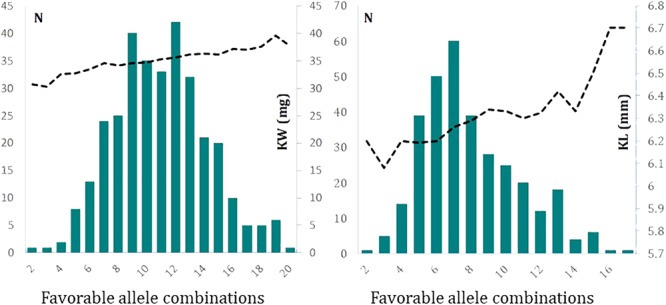
The frequency distribution of combinations of favorable alleles with their cumulative effects on determination of kernel weight **(Left)** and kernel length **(Right)**. The data used to produce these graphs are based on 2-year BLUP values. The left *y*-axis of each graph represents the frequency of lines and the right *y*-axis of graphs represent kernel weight in mg **(Left)** and kernel length in mm **(Right)**.

We were also interested to see up to how many favorable alleles are naturally present in a given germplasm. To do this, we counted the number of germplasm that accumulated from the lowest to the highest number of favorable alleles in the association panel. The frequency distribution of number of favorable alleles identified for KW in the germplasm followed a normal distribution (**Figure 5**). For the 26 identified loci for KW, we found lines with a minimum of two favorable alleles and lines with a maximum of 20 favorable alleles. Majority of entries (91.0%) possessed 6–16 favorable alleles. KW increased clearly with the increase in the number of favorable alleles. Using KW1617 BLUP values, the mean KW of entries with up to five favorable alleles combined (*n* = 12) was 32.3 g while the mean KW1617 for entries with ≥16 favorable alleles combined (*n* = 27) was 37.8 g, a difference of about 5.5 mg.

### Commutative Effect of Identified Loci on KL

Similar to the procedure performed for KW, considering the 27 identified loci for KL, we found lines with a minimum of two favorable alleles combined to lines with a maximum of 17 favorable alleles combined. The majority of entries (94.4%) possessed 4–13 favorable alleles combined. Increases in the number of the combinations of favorable alleles clearly increased KL (**Figure 5**). Using KL1617 BLUP values, the mean KL of entries with up to five favorable alleles combined (*n* = 59) was 6.17 mm while the mean KL for entries with ≥12 favorable alleles combined (*n* = 42) was 6.41 mm, a difference of about 0.23 mm.

### Effect of Previously Known Loci/Genes

The *t*-test results of comparing KW and KL of lines homozygous for alternate alleles of KASP markers is shown in **Table 2**. Most of loci/genes tested did not show a significant effect on KW and KL of this specific population. Of the six grain-related KASP markers tested, *TaGW2* has shown to be significantly associated with KW (*p*-value < 0.001) while *TaSus2-2B* and *TaGS-D1* were significantly associated with KL, with *p*-values < 0.001 and 0.02, respectively. The plant height loci *Rht-B1* was significant (*p*-value < 0.05) for KL, where the wild-type tall allele was associated with longer KL. The Mercia allele at the *Ppd-D1* locus has been shown to be significant for KW (*p*-value < 0.05).

**Table 2:** Effects of allelic variation of previously reported agronomic loci/genes on kernel weight and kernel length in the current mapping panel.

KASP assay	Frequency (variant)	Kernel weight			Kernel length		
		Mean AA	Mean BB	*p*-value	Mean AA	Mean BB	*p*-value
*Rht-B1*	231 (Rht-B1a)/88 (Rht-B1b)	35.36	36.38	0.1015	6.28	6.19	0.0364
*Rht_B1a_160IND*	205 (Rht-B1a)/116 (Rht-B1a+160)	35.59	35.56	0.9421	6.24	6.28	0.3506
*Rht_B1_197IND*	315 (Rht-B1a)/8 (Rht-B1a+197)	35.66	34.36	0.3451	6.25	6.46	0.1101
*Rht-D1*	276 (Rht-D1a)/45 (Rht-D1b)	35.8	34.79	0.1211	6.26	6.25	0.9481
*Ppd-A1*	256 (Ppd-A1a)/53 (Ppd-A1a.1_insens)	35.42	36.18	0.2588	6.26	6.27	0.8326
*Ppd-D1-Ciano67*	271 (Ppd-D1a)/37 (Ppd-D1a_Ciano67_insens)	35.51	36.02	0.5506	6.27	6.26	0.9127
*Ppd-D1-Mercia*	269 (Ppd-D1a)/47 (Mercia_type_insertion)	35.75	34.38	0.0188	6.25	6.29	0.4281
*Ppd-D1-Norstar*	130 (Ppd-D1a)/189 (Norstar_type_deletion)	35.99	35.40	0.2437	6.24	6.27	0.5413
*TaSus2-2B*	85 (TaSus2-2B)/226 (no TaSus2-2B)	35.32	35.66	0.5634	6.15	6.31	0.0003
*TaCWI-4A*	221 (Hap-4A-C)/88 (Hap-4A-T)	35.49	35.80	0.5750	6.25	6.30	0.2532
*TaTGW6-A1*	171 (TaTGW6-A1b)/143 (TaTGW6-A1b)	35.52	35.56	0.9400	6.25	6.27	0.5466
*TaGS-D1*	108 (TaGS-D1a)/199 (TaGS-D1b)	35.43	35.41	0.9661	6.33	6.23	0.0200
*TaGW2*	305 (TaGW2)/16 (TaGW2_SS-MPV57)	35.78	32.14	0.0006	6.25	6.37	0.3133

### Candidate Gene Identification

The annotated wheat reference genome was used to pull out high confidence protein-coding genes that are in the vicinity (±250 kb) of the polymorphic sites. This gene search has resulted in a total of 258 genes for KW (**Supplementary Table S7**) and 235 genes for KL (**Supplementary Table S8**). A short list of identified genes is categorized into functional groups of (1) cell cycle related genes, (2) carbohydrate metabolism and transport, (3) nitrogen metabolism and transport, (4) cell wall, (5) plant hormones, (6) post-translation modifications such as ubiquitination, and (7) seed maturation and biological events that resemble stress responses (**Tables 3, 4**).

**Table 3:** Candidate genes within the identified regions controlling kernel weight and their putative physiological roles.

QTL loci	Gene	Protein	Function	Reference
*QKWpur-2B.1*	TraesCS2B01G034100	Glycosyltransferase	Role in the biosynthesis of oligosaccharides, polysaccharides, and glycoconjugates	Breton et al., 2006; Lairson et al., 2008
*QKWpur-2D.1*	TraesCS2D01G020800	Photosystem II reaction center protein K	Photosynthesis	Vinyard et al., 2013; Caffarri et al., 2014
*QKWpur-2D.1*	TraesCS2D01G020900	Photosystem II reaction center protein I	Photosynthesis	Vinyard et al., 2013; Caffarri et al., 2014
*QKWpur-2D.1*	TraesCS2D01G021000	Photosystem II D2 protein	Photosynthesis	Vinyard et al., 2013; Caffarri et al., 2014
*QKWpur-2D.1*	TraesCS2D01G020200	Apyrase	Role in regulating growth and development	Riewe et al., 2008
*QKWpur-2D.2*	TraesCS2D01G141100	E3 Ubiquitin ligase family protein	Role in ubiquitin pathway	Li and Li, 2014
*QKWpur-3B.1*	TraesCS3B01G582000	Histone-lysine *N*-methyltransferase	Epigenetic regulation of expression (changes in DNA methylation or histone modification states)	Pontvianne et al., 2010
*QKWpur-3B.4*	TraesCS3B01G598100	Pectinesterase	Cellular adhesion and stem elongation	Micheli, 2001
*QKWpur-3B.4*	TraesCS3B01G597100	Phosphoenolpyruvate carboxykinase (ATP)	photosynthetic CO_2_-concentrating mechanisms of C4 photosynthesis [9] and crassulacean acid metabolism	Leegood and Walker, 2003
*QKWpur-3B.4*	TraesCS3B01G598200	Glycosyltransferase	Role in the biosynthesis of oligosaccharides, polysaccharides, and glycoconjugates	Breton et al., 2006; Lairson et al., 2008
*QKWpur-3B.4*	TraesCS3B01G595200	RING/U-box superfamily protein	Role in ubiquitin pathway	Yee and Goring, 2009
*QKWpur-3B.4*	TraesCS3B01G595400	Embryogenesis transmembrane protein-like	Involve in hormone transport system active during embryogenesis	Jahrmann et al., 2005
*QKWpur-4A.2*	TraesCS4A01G028000	Pectinesterase	Cellular adhesion and stem elongation	Micheli, 2001
*QKWpur-4A.3*	TraesCS4A01G440500	Protein nrt1 ptr family 1.2	Nitrate transporters in plants: structure, function and regulation	Forde, 2000
*QKWpur-4A.3*	TraesCS4A01G440600	Protein nrt1 ptr family 1.2	Nitrate transporters in plants: structure, function and regulation	Forde, 2000
*QKWpur-4A.3*	TraesCS4A01G440700	Protein nrt1 ptr family 1.2	Nitrate transporters in plants: structure, function and regulation	Forde, 2000
*QKWpur-4B*	TraesCS4B01G193000	6-phosphofructo-2-kinase/fructose-2, 6-bisphosphatase	Sucrose biosynthesis	Lunn, 2016
*QKWpur-5A*	TraesCS5A01G024700	Protein FANTASTIC FOUR 3	Potential to regulate shoot meristem size	Wahl et al., 2010
*QKWpur-7A.1*	TraesCS7A01G468200	SAUR-like auxin-responsive protein family	Role in auxin-mediated cell elongation	Jain et al., 2006
*QKWpur-7B.1*	*TraesCS7B01G082500*	*O-fucosyltransferase family protein*	Role in cell-to-cell adhesion	Verger et al., 2016

**Table 4:** Candidate genes within the identified regions controlling kernel length and their putative physiological roles.

QTL loci	SNP	Gene	Protein	Function	Reference
*QKLpur-1D*	S1D_445262848	TraesCS1D01G363700	Beta-galactosidase	Regulate cytokinins	Song et al., 2010
*QKLpur-2A.2*	S2A_719213280	TraesCS2A01G483000	Glycosyltransferase	Role in the biosynthesis of oligosaccharides, polysaccharides, and glycoconjugates	Breton et al., 2006; Lairson et al., 2008
*QKLpur-3A.1*	S3A_593313534	TraesCS3A01G343800	Photosystem I reaction center subunit VIII	Photosynthesis	Vinyard et al., 2013; Caffarri et al., 2014
*QKLpur-3A.2*	S3A_700575251	TraesCS3A01G467300	E3 ubiquitin-protein ligase BRE1-like 2	Role in ubiquitin pathway	Li and Li, 2014
*QKLpur-3A.2*	S3A_700575251	TraesCS3A01G467000	Late embryogenesis abundant (LEA) protein	Role in desiccation tolerance	
*QKLpur-3A.3*	S3A_700575251	TraesCS3A01G469200	Late embryogenesis abundant (LEA) protein	Role in desiccation tolerance	
*QKLpur-6A*	S6A_131449965	TraesCS6A01G149200	Ubiquitin-conjugating enzyme	Role in ubiquitin pathway	Li and Li, 2014
*QKLpur-6D*	S6D_436639209	TraesCS6D01G334300	Protein pelota homolog	Role in meiotic cell division	Eberhart and Wasserman, 1995; Caryl et al., 2000
*QKLpur-7A.3*	S7A_691163936	TraesCS7A01G501600	RING/U-box superfamily protein	Role in ubiquitin pathway	Yee and Goring, 2009

## Discussion

Much of the genetic gains for GY has been attributed to the increases in GN, while KW generally remained unchanged if not decreased (Sayre et al., 1997; Brancourt-Hulmel et al., 2003; Carver, 2009; Hawkesford et al., 2013). We could not conclude a definitive trend for KW and KL over the breeding history. Though a long-standing belief that correlation of GN and KW is negative, Miralles and Slafer (1995) and Acreche and Slafer (2006) argued that this negativity is not due to competition between grains. That means, it is possible to develop progeny with high KW and GN concurrently by carefully selecting parents, as was evidenced by the work of Bustos et al. (2013). Therefore, there may exist an untapped potential in KW to improve GY if given due consideration in the variety development process. While further increases in GY can be dependent on maintaining, if not increasing, KW, an alternative breeding strategy could be to increase KW while maintaining GN or increasing KW and GN simultaneously. Careful recycling of high KW accessions including those developed before 1920 could improve kernel traits and ultimately result in gains in GY.

In this study, we detected 26 regions for KW and 27 regions for KL on most of the chromosomes, indicating that these traits are controlled by a complex genetic system. Previously, a large number of QTL for KW and dimension traits (kernel length, width, and thickness) have been reported across all 21 chromosomes of wheat (McCartney et al., 2005; Röder et al., 2008; Jiang et al., 2011; Bednarek et al., 2012; Deol et al., 2013; Simmonds et al., 2014; Hanif et al., 2015; Jiang et al., 2015; Su et al., 2016). Our evaluation of some of the previously reported genes and related functional markers like Kompetitive Allele Specific PCR (KASP) markers for kernel-related traits revealed that most of them had no significant effect of KW and KL in this panel. The exceptions were *TaGW2* for KW; and *TaSus2-2B* and *TaGS-D1* for KL. The non-significant effect for most of the loci may be that these genes are background dependent, inviting further evaluation of the effect of these genes in the different genetic background.

Kernel weight, as one of the main GY determinant (Simmonds et al., 2014), holds a very high heritability, reaching to *h*^2^ = 87% (Bergman et al., 2000). In the current study, we also reported high heritability estimates of 61% for KW and 55% for KL. In allele enrichment schemes, breeders usually work to increase the frequency of favorable alleles. Our data suggest that favorable alleles at *QKW_pur_-3B.1, QKW_pur_-4A.1, QKW_pur_-4A.2*, and *QKW_pur_-5B.1* having low frequencies (3–9%) in germplasm released after 2000 and are prospect targets of selection for KW improvement. Similarly, loci *QKL_pur_-2A.1, QKL_pur_-2D, QKL_r_-3A.2, QKL_pur-_3A.3, QKL_pur_-3A.4, QKL_pur_-4D and QKL_pur-_6B* could be potential targets for breeding via enriching the favorable allele frequency in the current breeding populations.

Wheat lags diploid model plants such as rice and Arabidopsis for the availability of genome-wide resources and tools. Recently, mutant resources in tetraploid and hexaploid wheat have become available^4^. In addition, the wheat reference genome IWGSC RefSeq v1.0 annotation v1.0^5^ (see footnote 2) made it possible to connect next-generation sequencing-based markers to candidate gene identification in GWAS studies using a position-dependent strategy. In our study, we assessed the genes within 250 kb of the QTL loci and listed potential candidate genes.

Kernels that have the potential for growth and are well filled during grain-fill period weigh more (Jenner et al., 1991; Altenbach and Kothari, 2004). A fine component of sink-strength is grain enlargement, which is enforced by endosperm cell division followed by water uptake (Jenner et al., 1991; Emes et al., 2003; Altenbach and Kothari, 2004). Source-strength, on the other hand, is an expression of supply of assimilates, i.e., starch and storage protein through current photosynthesis or remobilization of reserves from vegetative tissues (Bidinger et al., 1977; Schnyder, 1993; Gebbing and Schnyder, 1999). The conceptual framework for grain development may involve processes such as cell division, enlargement, and embryogenesis; photosynthesis, carbohydrate metabolism, and nitrogen metabolism; and post-translational modifications. Thus, our discussion for candidate genes for KW and KL concentrate on genes involved in the above-mentioned processes.

Grain enlargement commences with fertilization, wrapped-up within about 20 days after fertilization, and it also coincides with the period of mitotic activity (Jenner et al., 1991), as was observed in this study. The association with the largest signal [*–log_10_(p)* = 5.4] was *QKW_pur_-7B.1* and this locus was found within 107 kb from *TraesCS7B01G082500*, which codes for *O-fucosyltransferase family protein* (**Table 3**). This protein was reported to have a function in cell-to-cell adhesion during plant growth and development (Verger et al., 2016). The gene *TraesCS3B01G595400* was in proximity of *QKW_pur_-3B.4* [*–log_10_(p)* = 3.8] and encodes an embryogenesis transmembrane protein-like (**Table 3**). Jahrmann et al. (2005) highlighted that an embryogenesis transmembrane protein involved in hormone transport during embryogenesis. The *TraesCS5A01G024700* encoding for a *FANTASTIC FOUR 3* was associated with *QKW_pur_-5A* [*–log_10_(p)* = 3.6], is potentially involved in regulating shoot meristem size (Wahl et al., 2010). A *SAUR-like auxin-responsive* protein family (*TraesCS7A01G468200*) that we show it to be associated with *QKW_pur_-7A.1* [*–log_10_(p)* = 3.6], may have a role in auxin-mediated cell elongation (Jain et al., 2006). The *QKLpur-6D* [*–log_10_(p)* = 4.1] is within ±250 kb of *TraesCS6D01G334300*, a gene that encodes for protein pelota homolog (**Table 4**), previously reported to have a role in meiotic cell division (Caryl et al., 2000).

Kernel development is wrapped up by maturation. Tang et al. (2016) indicated that late embryogenesis abundant (LEA) genes become abundant during the late stages of seed development and enable the maturing seeds to acquire the desiccation tolerance. Temporal differences in expression of these genes may be a good signal for differences in the arrest of enlargement of the growing kernels. Two loci responsible for KL, i.e.; *QKL_pur_-3A.2* [*–log_10_(p)* = 3.8] and *QKL_pur_-3A.3* [*–log_10_(p)* = 4.1] were linked to wheat genes TraesCS3A01G467000 and TraesCS3A01G469200, which are predicted to encode *late embryogenesis abundant protein* (**Table 4**).

The QTL on 2D, *QKW_pur_-2D.1* [*–log_10_(p)* = 3.7], was found to be associated with *Apyrase* (**Table 3**). Riewe et al. (2008) silenced *apyrase* gene in potato using RNAi that led to less than 10% Apyrase activity. This ultimately changed the phenotypes in transgenic lines, including a general retardation in growth, an increase in tuber number per plant, and differences in tuber morphology.

Three genes *TraesCS2D01G020800, TraesCS2D01G020900*, and *TraesCS2D01G021000* encoding photosystem reaction center proteins were found near *QKW_pur_-2D.1* with *–log_10_(p)* = 3.7 (**Table 3**). The photosystem II is the reaction center that uses light energy to split water into hydrogen and oxygen, and release electrons that will be transferred to the second photosynthetic reaction center called photosystem I (Caffarri et al., 2014). We also identified a gene which encodes for photosystem I reaction center subunit VIII (*TraesCS3A01G343800*) and is within ±250 kb of *QKL_pur_-3A.1*, with *–log_10_(p)* = 3.6 (**Table 4**). As current assimilates filling the developing kernels are direct products of photosynthesis, the candidacy of these photosystem reaction proteins seems to be logical and is worth validation studies.

Starch accumulation accounts for 60–75% of kernel dry matter and mainly responsible for kernel size and yield (Rahman et al., 2000; De Gara et al., 2003). Sucrose is the most common form of carbohydrate transported from source to sink organs. Thirty-eight kilo base away from *QKW_pur_-4B* [*–log_10_(p)* = 4.5], we identified *TraesCS4B01G193000* which encodes a *fructose-2,6-bisphosphatase* (**Table 3**) that is involved in the dephosphorylation step of *sucrose synthesis* (Lunn, 2016). Transgenic Arabidopsis plants with only 5% *fructose-2,6-bisphosphates* expression, as compared to wild-type plants, demonstrate altered partitioning of carbon between sucrose and starch (Draborg et al., 2001). McCormick and Kruger (2015) reported that the T-DNA insertional Arabidopsis mutant lines for *fructose-2,6-bisphosphates* showed reduced growth and seed yields compared with wild-type plants. This enzyme was also reported to play a role in the partitioning of photoassimilate in sorghum (Reddy, 1996) and wheat (Reddy, 2000).

A QTL was reported previously that enhances KW and GY in rice via increases in cell numbers, allowing grains to reach to higher potential sizes. This QTL, named *GW2* in rice, was found to be a *RING-type protein E3 ubiquitin ligase* activity, with loss of function mutant (Song et al., 2007). Our study resulted in identification of two loci, i.e.; *QKWpur-2D.2* [*–log_10_(p)* = 3.7] and *QKL_pur_-3A.2* [*–log_10_(p)* = 3.8] that are associated with *E3 ubiquitin-protein ligase* via *TraesCS2D01G141100* and *TraesCS3A01G467300*, respectively (**Tables 3, 4**).

## Conclusion

This study utilized genome-based markers and resulted in the identification of loci and genes important to the determination of grain traits. We have also demonstrated that GWAS results can be utilized to further investigate genomic regions to drive putative list of candidate genes that can be further validated. The immediate use of this data could be developing breeder friendly markers (i.e., KASP) that can be useful in breeding. Further functional genomic studies are crucial to validate the effect of the identified candidate genes on KW and dimension traits. Utilizing mutant resources developed recently (Krasileva et al., 2017) is one way to functionally validate the effect of these candidate genes in the determination of KW and KL.

## Data Availability

The genotypic and phenotypic data pertaining to the analysis and conclusion are available via the link: https://de.cyverse.org/de/?type=data&folder=/iplant/home/shared/commons_repo/staging/Daba_KernelTriats_2018.

## Author Contributions

GB-G and PT executed genome-wide marker development at the Small Grains Genotyping Laboratory at USDA-ARS in Raleigh, NC, United States and participated in the writing of the manuscript. MM and SD designed the study, collected all the data, performed all the statistical and blast analysis, and wrote the manuscript. SD also conducted the SNP calling using IWGSv1.0.

## Conflict of Interest Statement

The authors declare that the research was conducted in the absence of any commercial or financial relationships that could be construed as a potential conflict of interest. The reviewer JC and handling Editor declared their shared affiliation.

## Footnotes

1 https://bitbucket.org/tasseladmin/tassel-5-source/wiki/Tassel5GBSv2Pipeline

2 https://wheat-urgi.versailles.inra.fr/Seq-Repository/Assemblies

3 https://urgi.versailles.inra.fr/download/iwgsc/IWGSC_RefSeq_Annotations/v1.0/

4 http://www.wheat-tilling.com/

## Supplementary Material

FIGURE S1 Phenotypic distributions of kernel weight (top) and kernel length (bottom) measured in 2016 (left) and 2017 (right).

FIGURE S2 Scatterplot showing correlation of BLUP values of KW and KL over the two years of study.

FIGURE S3 Changes over the four year-groups (1 = before 1920, 2 = 1920 to 1960, 3 = 1960 to 2000, and 4 = after 2000) observed in kernel weight (top) for measurements in 2016 **(a)**, 2017 **(b)**, and for the BLUP values across the two years of study **(c)**; and in kernel length (bottom) for measurements in 2016 **(d)**, 2017 **(f)**, and the BLUP values across the two years of study **(f)**.

FIGURE S4 Plots of *F*_ST_ statistics for pairs of sub-populations generated using the model-based clustering procedure.

FIGURE S5 Manhattan plots showing negative log *p*-values of SNPs tested across the 21 chromosomes (i.e., 1 = 1A, 2 = 1B, 3 = 1D, ..., 20 = 7B, and 21 = 7D) for kernel weight (top) and kernel length (bottom) for traits measured in 2016 (left) and 2017 (right).

FIGURE S6 Frequency of favorable alleles observed in each of the year-group for a selected number of loci controlling kernel weight.

FIGURE S7 Frequency of favorable alleles observed in each of the year-group for a select number of loci controlling kernel length.

TABLE S1 The accuracy of imputation at different levels of marker masking using LDKNNi procedure in TASSEL. We used 30 sites for LD estimation. The number of nearest neighbors of entries was 10.

TABLE S2 Cluster membership of the 324 genotypes used in model-based clustering with the year in which the accession was registered at NSGC.

TABLE S3 The GWAS statistics for each marker-trait association for kernel weight in 2016, 2017, and combined year data. The table includes variants, minor allele frequency (MAF), -logP, R2, and allelic effect.

TABLE S4 The GWAS statistics after categorizing MTAs into QTL regions kernel weight in 2016, 2017, and combined year data. The table includes variants, minor allele frequency (MAF), -logP, R2, and allelic effect.

TABLE S5 The GWAS statistics for each marker-trait association for kernel length in 2016, 2017, and combined year data. The table includes variants, minor allele frequency (MAF), -logP, R2, and allelic effect.

TABLE S6 The GWAS statistics after categorizing MTAs into QTL regions kernel length in 2016, 2017, and combined year data. The table includes variants, minor allele frequency (MAF), -logP, R2, and allelic effect.

TABLE S7 The putative candidate genes found nearby the polymorphic sites for kernel weight.

TABLE S8 The putative candidate genes found nearby the polymorphic sites for kernel length.
