# Supplementary material for: Genome-Wide Association Studies to Identify Loci and Candidate Genes Controlling Kernel Weight and Length in a Historical United States Wheat Population
Source: Front Plant Sci. 2018 Aug 3;9:1045. doi: 10.3389/fpls.2018.01045 (PMC6086202; doi:10.3389/fpls.2018.01045)
Supplement: FIGURE S1 — Phenotypic distributions of kernel weight (top) and kernel length (bottom) measured in 2016 (left) and 2017 (right). [file Data_Sheet_1.docx]

**Supplemental Figures**

**Figure S1**. Phenotypic distributions of kernel weight (top) and kernel length (bottom) measured in 2016 (left) and 2017 (right)*.*

**
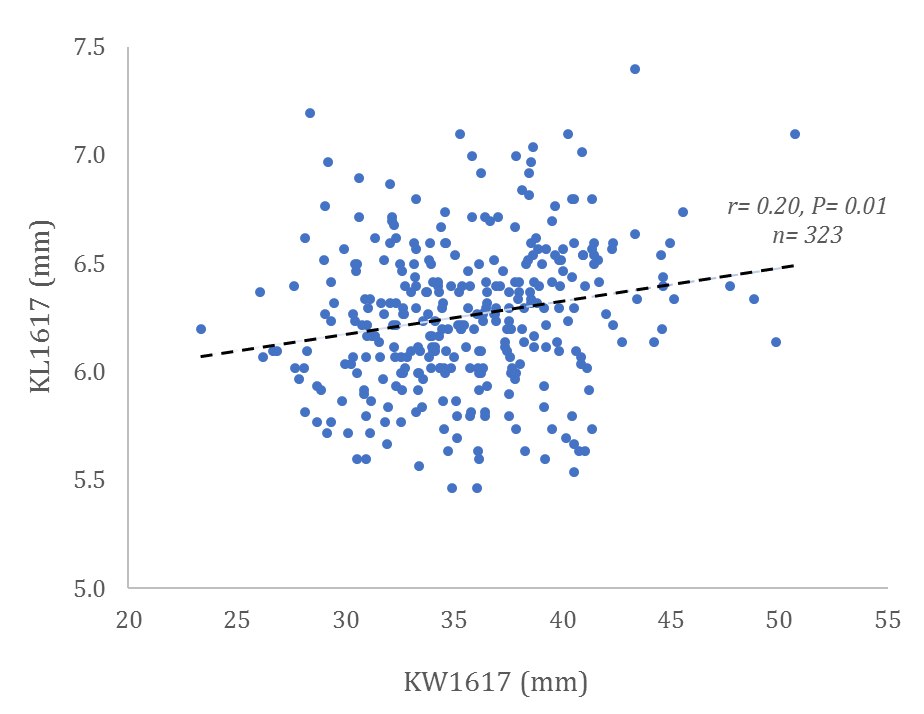
**

**Figure S2**. Scatterplot showing correlation of BLUP values of KW and KL over the two years of study.

**Figure S3.** Changes over the four year-groups (1 = before 1920, 2= 1920 to 1960, 3 = 1960 to 2000, and 4 = after 2000) observed in kernel weight (top) for measurements in 2016 (a), 2017 (b), and for the BLUP values across the two years of study (c); and in kernel length (bottom) for measurements in 2016 (d), 2017 (f), and the BLUP values across the two years of study (f).

Fst

Fst

Fst


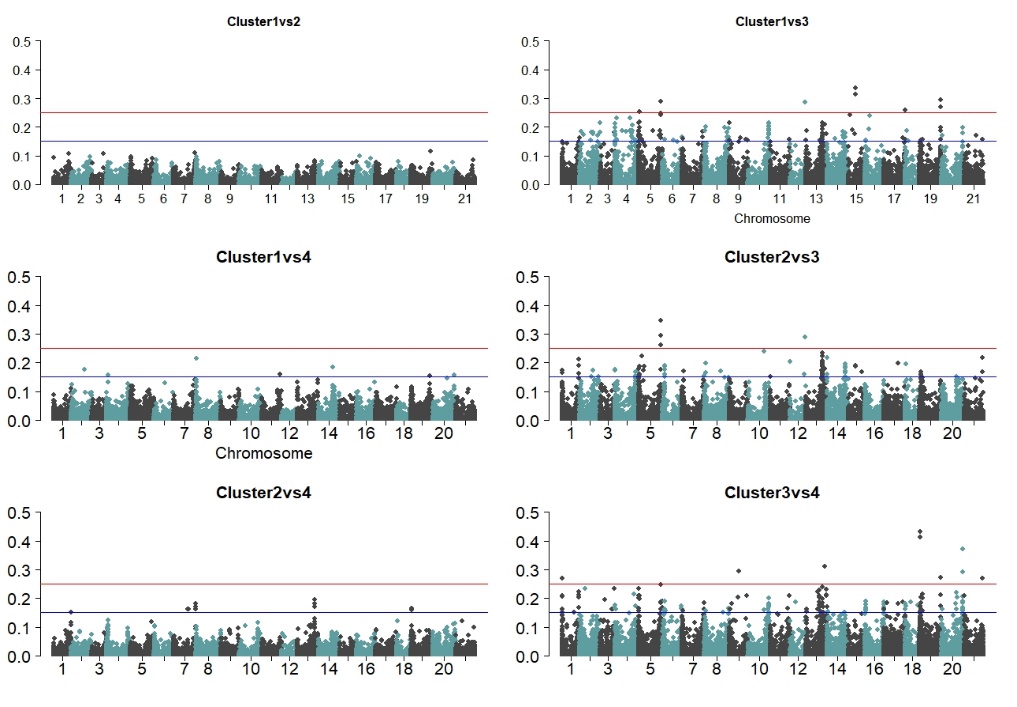


**Figure S4.** Plots of*F_ST_* statistics for pairs of sub-populations generated using the model-based clustering procedure.

*-log_10_P*

*-log_10_P*


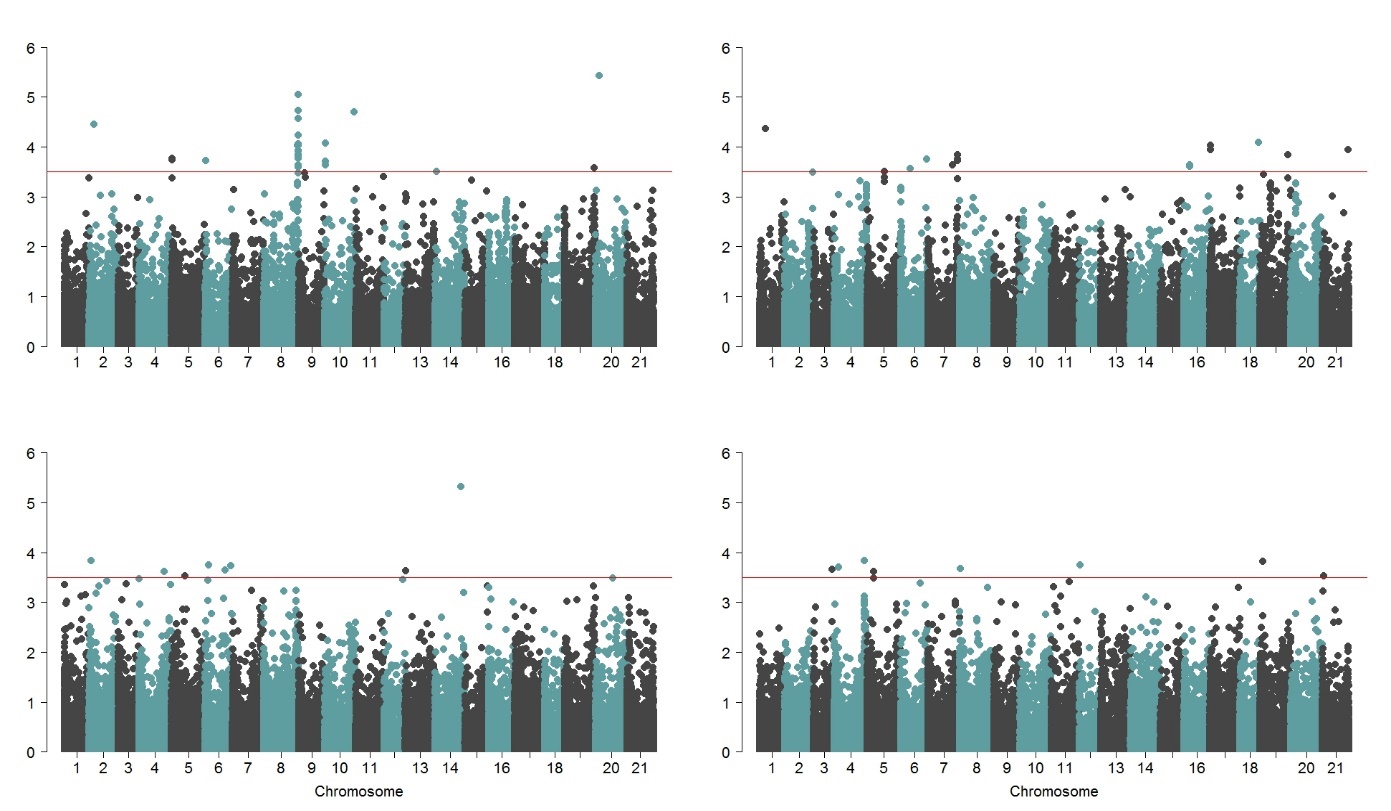


**Figure S5**. Manhattan plots showing negative log p-values of SNPs tested across the 21 chromosomes (i.e., 1= 1A, 2= 1B, 3 = 1D, …, 20 = 7B, and 21 = 7D) for kernel weight (top) and kernel length (bottom) for traits measured in 2016 (left) and 2017 (right).


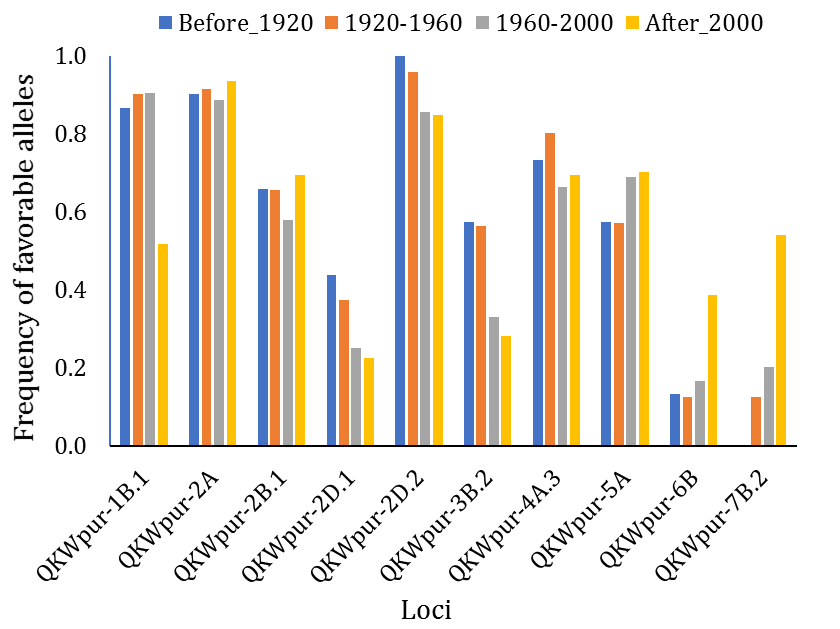


**Figure S6.** Frequency of favorable alleles observed in each of the year-groupfora selected number of loci controlling kernel weight.


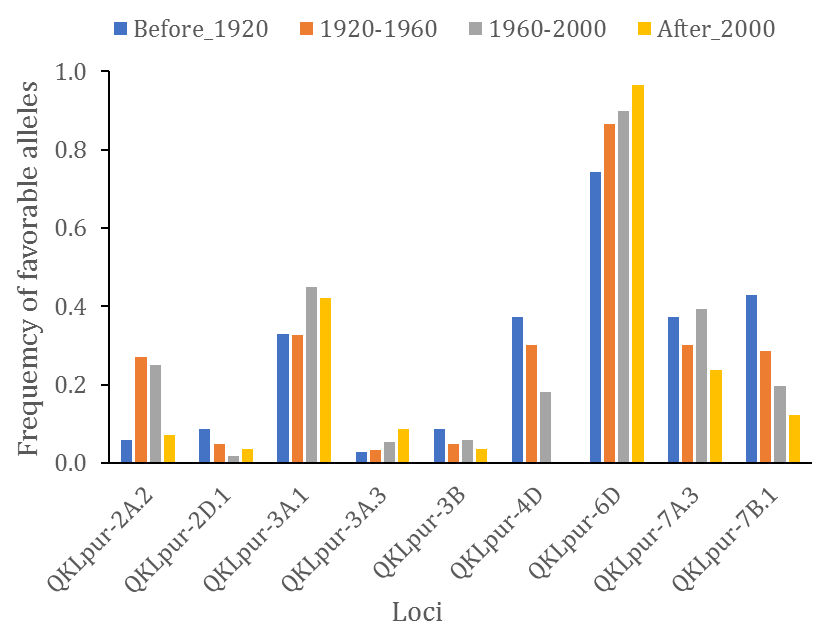


**Figure S7.** Frequency of favorable alleles observed in each of the year-group fora select number of loci controlling kernel length.
